# Drivers of differences in expected public health impact of RTS,S in individual countries of Africa: an individual-based modeling study

**DOI:** 10.1186/1475-2875-13-S1-P68

**Published:** 2014-09-22

**Authors:** Melissa Penny, Katya Galactionova, Thomas Smith

**Affiliations:** 1 Swiss Tropical and Public Health Institute, Basel, Switzerland; 2 University of Basel, Basel, Switzerland

## Background

RTS,S is a malaria vaccine directed at the pre-erythrocytic stage of *Plasmodium falciparum*, and is currently in late-stage Phase III clinical trials at multiple sites across Africa. Modeling and simulation have been used to predict the public health impact (PHI) of RTS,S but previous exercises had access to limited trial data and did not provide country-specific results. PHI is sensitive to many assumptions including vaccine profile and underlying country specific inputs.

## Materials and methods

We simulated the numbers of cases and deaths averted by RTS,S if rolled out in each of 43 malaria endemic countries in sub-Saharan Africa. Predictions were made for different transmission profiles, different levels of access to care for malaria cases, and four different immunisation schedules, with varying initial efficacies and decay rates of vaccine efficacy. Country-specific predictions were made via weighted averages over the database of predictions, with weights estimated from country-specific data. These included prevalence data from the Malaria Atlas Project, access to care from Demographic and Health Surveys, country level estimates of patient adherence and system compliance, immunization coverage from UN/WHO, and demographic projections from the UN. Sensitivity analysis was carried out to determine the robustness of the predictions of average impact and main drivers of uncertainty in these.

## Results and conclusions

A pre-erythrocytic vaccine is unlikely to completely protect vaccinated individuals against malaria disease but overall PHI will probably compare favourably with that of other vaccines in routine programs. Currently only short follow up of RTS,S Phase III trial data is available and thus uncertainty in overall PHI remains mainly driven by uncertainty in the vaccine profile, especially in the rate of decay of the protective effects. There will be considerable variation between countries in PHI, with the main driver of this variation being the underlying levels of transmission and disease burden.

